# Electroanalytical Approaches to Combatting Food Adulteration: Advances in Non-Enzymatic Techniques for Ensuring Quality and Authenticity

**DOI:** 10.3390/molecules30040876

**Published:** 2025-02-14

**Authors:** Fotios Tsopelas

**Affiliations:** Laboratory of Inorganic and Analytical Chemistry, School of Chemical Engineering, National Technical University of Athens, Zografou, 157 72 Athens, Greece; ftsop@central.ntua.gr

**Keywords:** food fraud, adulteration of foodstuffs, non-enzymatic electroanalytical techniques, voltammetry, chemometrics, Principal Component Analysis, class-modeling, machine learning applications

## Abstract

Food adulteration remains a pressing issue, with serious implications for public health and economic fairness. Electroanalytical techniques have emerged as promising tools for detecting food adulteration due to their high sensitivity, cost-effectiveness, and adaptability to field conditions. This review delves into the application of these techniques across various food matrices, including olive oil, honey, milk, alcoholic beverages, fruit juices, and coffee. By leveraging methodologies such as voltammetry and chemometric data processing, significant advancements have been achieved in identifying both specific and non-specific adulterants. This review highlights novel electrodes, such as carbon-based electrodes modified with nanoparticles, metal oxides, and organic substrates, which enhance sensitivity and selectivity. Additionally, electronic tongues employing multivariate analysis have shown promise in distinguishing authentic products from adulterated ones. The integration of machine learning and miniaturization offers potential for on-site testing, making these techniques accessible to non-experts. Despite challenges such as matrix complexity and the need for robust validation, electroanalytical methods represent a transformative approach to food authentication. These findings underscore the importance of continuous innovation to address emerging adulteration threats and ensure compliance with quality standards.

## 1. Introduction

The globalization of our food supply has amplified various risks, including economically motivated adulteration (EMA) and food fraud. EMA involves altering the characteristics of food or partially substituting it with less expensive materials (e.g., seed oils in olive oil or sugar syrups in honey) or entirely inexpensive components (e.g., water) to maximize profits. Commonly targeted foods and ingredients include oils, fish, honey, milk and dairy products, meat products, grain-based foods, fruit juices, wine, alcoholic beverages, organic foods, spices, coffee, tea, and certain highly processed foods [1]. Some adulterants, like water in liquid products, are added to increase volume, while others, such as sweetening agents, are used to maintain or improve quality [2].

Food fraud also encompasses misrepresentation through the misbranding or mislabeling of the manufacturer, geographical origin, and cultivation practices [3,4,5,6,7]. Common examples include falsely labeling conventional food as “organic”, marketing inexpensive wines as premium brands or rare vintages, and mispresenting a product’s geographical origin when foods from certain regions command higher market prices (e.g., Prosciutto di Parma (Italy), Fava “Santorinis” (Greece), and Hierbas de Mallorca (Spain)). Detecting and quantifying food fraud and adulteration is critical for ensuring compliance with legal requirements, international standards, and directives; protecting public health; and safeguarding producers and farmers from unfair competition. In Table 1, the most common adulterants for representative foodstuffs and beverages are presented. In some cases, adulterants can lead to serious adverse effects on consumers, such as the addition of hydrogen peroxide, melamine, and urea in milk [8]. However, the task is analytically challenging due to the complexity of different food matrices, the wide range of adulterants, many of which exhibit similar physicochemical properties, as well as the need to minimize analysis time and costs and facilitate field analyses.

Electroanalytical techniques offer a popular alternative. These methods are based on the interaction between analytes and an electrode within an electrochemical cell. By measuring electrical properties such as current, voltage, or charge, the composition or the concentration of a certain analyte in a solution can be determined. The working electrode, where the analyte interacts, is the key component of any electroanalytical system. A reference electrode provides stable potential, while the counter/auxiliary electrode completes the electrical circuit. Electroanalytical techniques include potentiometry, which measures the potential between a working and a reference electrode at zero current (no reaction) and amperometry, which measures the current at a fixed potential and is usually combined with an appropriate biological recognition element (e.g., an enzyme) to develop biosensors and electrochemical impedance spectroscopy (EIS) measuring the impedance of a system in response to an alternating current signal. The most useful electroanalytical technique is voltammetry, which can be further classified as cylic voltammetry (CV), differential pulse voltammetry (DPV), square wave voltammetry (SQW), and stripping voltammetry [9,10]. Electroanalytical techniques offer significant advantages for food authenticity verification due to their high sensitivity, simplicity, low cost, miniaturization potential, and suitability to be used under field conditions.

Despite these advantages, a systematic review of non-enzymatic electroanalytical techniques for detecting food adulteration is still lacking. This review focuses on the adulteration of foodstuffs that are frequently targeted, including olive oil, honey, milk and dairy products, and alcoholic beverages, using non-enzymatic electroanalytical techniques. It does not address issues related to the geographical origin of products or the detection of harmful substances such as agrochemicals.

**Table 1:** Common adulterations in foodstuffs and beverages.

Foodstuff/Beverage	Adulterants	Reference
Olive oil	Seed oils (e.g., sunflower, soybean, sesame, corn, and hazelnut oil), olive oil of lower grade (e.g., olive pomace oil, lampante olive oil).	[1,11]
Honey	Sugar, rice syrups, barley syrups, corn syrups, rice molasses, and less expensive honey (e.g., polyfloral).	[2,12,13]
Milk and dairy products	Water, starch, glucose and other sugars, soybean and pea protein isolates, boric acid, salicylic acid, benzoic acid, melamine, urea, maltodextrose, cheese whey (byproduct of cheese production), hydrogen peroxide, and reconstituted skim milk powder. Different milk species (cow’s, sheep’s, and buffalo’s milk).	[1,2,8,14]
Wine	Synthetic sweeteners (e.g., saccharin), sugar, ethanol, flavor, water, synthetic dyes, and apple juice.	[1,3,5,6,7]
Whiskey	Alcohol (non-drinking or cereal alcohol), water caramel, dyes, flavors, beverages of lower commercial value, whiskey of different brands, aging, and blending (lower cost).	[1,4]
Beer	Flavor, different brands, and fermentation type (lower cost).	[15]
Fruit juices	Dilution with water, addition of other, less expensive fruit juices (e.g., lemon and grape fruit juice in orange juice), glucose-fructose syrup, formaldehyde, artificial flavor agents and dyes (e.g., rhodamine B), and salicylic acid.	[16,17,18,19,20,21]
Coffee	Grains (e.g., soybean, corn, barley, rice, triticale, rye, and chicory), brown sugar, defective coffee beans, coffee processing byproducts (e.g., coffee husks, sticks, and used coffee grounds), and cheaper varieties (e.g., Robusta (Coffea canephora) in Arabica (Coffea arabica)).	[22,23]

## 2. Analytical Strategy for Detection/Quantification of Food Adulteration

Detecting and quantifying food adulteration is a challenging task that requires adopting an appropriate strategy. In simpler cases where a single analyte must be detected, various analytical methods—primarily chromatography coupled with suitable detectors—can be employed. However, most cases involve unknown adulterants or adulteration using substances with similar physicochemical properties (e.g., seed oils in olive oil or inexpensive honeys in high-value, polyfloral honey). In such situations, a fingerprinting strategy is commonly used, which involves creating an analytical fingerprint of authentic samples and comparing it with that of a suspect sample.

Two fingerprinting approaches can be employed:

▪ Specific Fingerprinting: This method measures the concentrations of specific analytes, such as the profiles of fatty acids, sterols, and triacylglycerols in olive oil [24], or rare earth element profiles in lentils [25].
▪ Non-Specific Fingerprinting: Instead of targeting specific analytes, this approach uses the entire analytical signal (e.g., spectrum or voltammogram) as a multivariate representation of the sample’s chemical composition. Non-specific fingerprints can be generated using techniques like Ultra-Violet (UV-Vis) Spectrometry [26,27], Fourier Transform Infrared Spectrometry (FT-IR) [28], Fluorescence Spectrometry [26], Mid-Infrared Spectroscopy (MIR) [29], Raman Spectrometry [26], Nuclear Magnetic Resonance (NMR) [26,30], chromatography [31], Mass Spectrometry [32], or even Differential Scanning Calorimetry [33].

These fingerprints are typically two-dimensional (2D), but they can also take the form of three-dimensional (3D) signals, such as surfaces or images (e.g., optical or thermal images, or chromatogram spectra). A key advantage of fingerprinting is its ability to detect unexpected changes in chemical composition—such as the addition of novel or multiple adulterants—without requiring a predefined hypothesis.

In most cases, the information embedded in the chemical fingerprint is not immediately apparent and requires further processing to extract meaningful insights. Data mining techniques are often applied to classify samples as authentic or adulterated and quantify the level of adulteration. Common chemometric techniques for sample grouping include Principal Component Analysis (PCA), discriminant methods such as Linear Discriminant Analysis (LDA), Support Vector Machines (SVM), and Partial Least Squares-Discriminant Analysis (PLS-DA) [34,35,36], as well as class-modeling methods like Soft Independent Modeling of Class Analogy (SIMCA) [34,37]. Discriminant methods are appropriate when at least two well-defined classes exist. However, many food authenticity cases (compliant vs. not-compliant samples) do not constitute a true two-class problem. This is because non-compliant samples do not form a distinct group; they may include expired or defective products, as well as samples adulterated with various substances/materials, some of which may be newly introduced. Class-modeling (or one-class classification) techniques, which model a specific class of authentic samples, are particularly effective for food authentication. These techniques answer the critical question: “Is this sample, claimed to belong to class X, truly consistent with the class X model?” [34,37]. The performance of classification methods is expressed mainly in terms of sensitivity (prevalence of true positive samples) and specificity (prevalence of true negatives), while accuracy (ratio of correct assignments considering the entire dataset) and inconclusive ratio (number of non-assigned samples divided by the number of samples belonging to the class) have also been proposed [38].

It should be noted that chemometric techniques possess some limitations. These include the requirement of a large number of samples to develop a reliable model, dependence on data quality (e.g., unbiased and representative samples), and the frequent need for data preprocessing (e.g., reducing the number of initial variables). Furthermore, selecting the most suitable chemometric technique for a given problem requires expertise to ensure model interpretability—understanding the relationships between variables and explaining the reasoning behind predictions. Additionally, overfitting must be avoided, as it can lead to poor generalization and unreliable predictions on new data [39]. Lastly, coding skills are essential for applying most chemometric techniques, as they are typically implemented using programming environments, such as R 4.4.2., Python 3.13, or MATLAB 2024b.

Electroanalytical techniques can also be employed for detecting and quantifying food adulteration. After suitable sample pretreatment (e.g., dissolution, dilution, filtration, liquid–liquid or solid-phase extraction, and electrolyte addition), a sample can be analyzed for specific adulterants (e.g., melamine in milk). Electrochemical signals can then be processed to enhance their informativeness, such as subtracting non-informative capacitive currents and isolating faradaic currents [40] or utilizing the first-order derivative of the measured current versus applied potential [41,42]. Quantification is achieved by constructing calibration plots.

**Figure 1:**
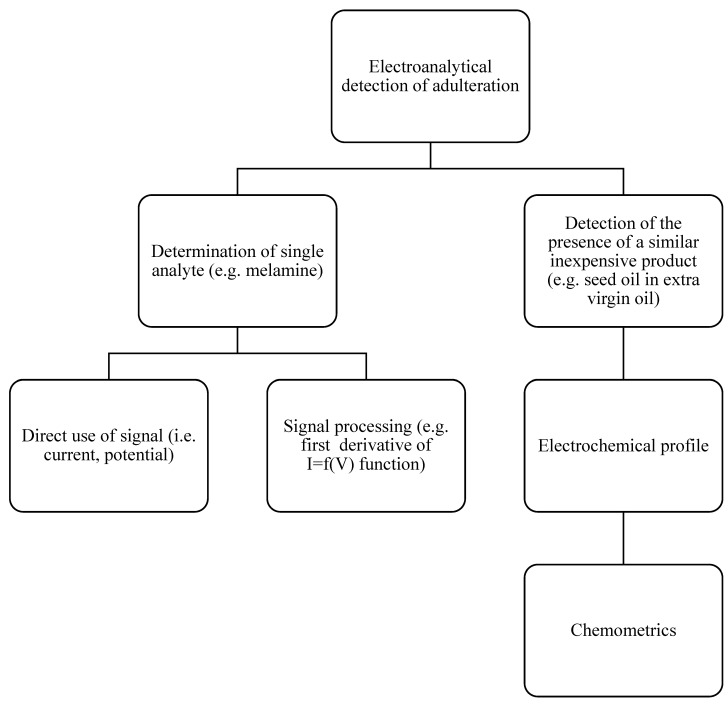
Strategy for the identification and quantification of food adulteration using electroanalytical techniques.

In cases where unknown adulterants have been added, the complete electrochemical graph (e.g., voltammogram) or a specific segment of it can serve as a non-specific multivariate description of the sample. Adulteration can then be identified and quantified using the chemometric techniques presented above. The electroanalytical workflow for identifying and quantifying food adulteration is illustrated in Figure 1.

## 3. Olive Oil

The voltammetric differentiation between extra virgin olive oil and refined olive oil is based on the fact that significant amounts of aromatic compounds, micronutrients, and antioxidants such as, polyphenols, tocopherols, sterols, and carotenoids are lost during the refining process [43]. Notably, the electrochemically active phenolic compounds are primarily responsible for the flavor of extra virgin olive oil, and serve as biomarkers of its authenticity [44]. More to the point, extra virgin olive oil contains high levels of phenolic compounds, which play a crucial role in its resistance to auto-oxidation and photo-oxidation. In contrast, refined olive oil, olive pomace oil, and seed oils contain substantially lower amounts of these compounds [45]. Alpha-tocopherol and squalene are also present in higher concentrations in extra virgin olive oil [46,47].

Electroanaytical measurements in oil matrices pose significant challenges due to very poor conductivity, the low solubility of many electrolytes in the oil phase, as well as incompatibility with the standard solvents (e.g., water, alcohols) typically used for electrochemical measurements. Hence, appropriate sample pretreatment is essential, such as dilution with appropriate electrochemically inert solvents that can dissolve small amounts of electrolytes. However, using low-polarity solvents shifts voltammetric peaks to higher potentials due to the increased energy required for electrochemical oxidation or reduction. This occurs because of the limited solvation of the polar products of the electrochemical process, also causing the voltammetric peaks to broaden or become indistinct [48]. An alternative approach involves solubilization within the hydrophobic cores of micro-heterogeneous media, such as micelles or emulsions [49].

An effective strategy was reported by the research group of Prof. P. Oliveri, involving the addition of room temperature ionic liquids (RTILs), such as the tri-hexyl(tetradecyl)phosphonium bis(trifluoromethylsulfonyl)imide. These RTILs enhance the conductivity of the matrix [49,50,51,52]. Oliveri et al. successfully distinguished olive oils from maize oils and classified olive oils by geographical origin using cyclic voltammograms recorded on platinum microelectrodes. The resulting voltammetric data were analyzed using the chemometric technique of K-nearest neighbors [49]. Additionally, Prof. P. Oliveri’s team employed the same ionic liquid to determine the free acidity of olive oils, employing a radius platinum microdisk working electrode. They analyzed current intensities at fixed potentials using chronoamperometry, or cyclic voltammetry using linear regression [51], or used the entire cyclic voltammetric profile, 0.0 and −1.4 V with PLS regression [52]. Notably, not all ionic liquids are suitable for such applications. Some can significantly increase the viscosity of oil samples, complicating preparation and handling. Additionally, the high cost of certain RTILs and their potential environmental impact due to limited biodegradability present challenges that need to be addressed.

Apetrei et al. proposed a novel methodology using oil samples as electroactive binders to fabricate carbon paste electrodes chemically modified with oils. Electroanalytical signals were recorded by immersing these electrodes in different aqueous electrolytic solutions [53,54]. Although the number of samples tested was limited, the approach effectively discriminated extra virgin olive oil from refined olive oils and seed oils [53]. A similar approach was employed by the same authors to evaluate the bitterness intensity of extra virgin olive oils, with results obtained by the panel of experts [54]. Apetrei and Apetrei further developed voltammetric E-tongues to detect olive oil adulteration with seed oils [55].

Tsopelas et al. introduced two different sample pretreatments for oil analysis using cyclic voltammetry on a glassy carbon electrode: the dilution of oils with dichloromethane and ethanol and extraction with methanol. Cyclic voltammetric data between 0.0 V and +1.3 V analyzed via PLS-DA revealed clear discrimination between olive oils (extra virgin and refined) and olive pomace/seed oils. Class-modeling further distinguished extra virgin olive from all other samples. When using methanolic extracts and analyzing data points between 0.6 and 1.3 V, PLS-DA produced three distinct clusters: extra virgin olive oils, regular olive oils, and seed/olive pomace oils. A PLS model was also developed to quantify extra virgin olive oil adulteration with olive pomace oil or seed oils, achieving a detection limit of 2% *v*/*v* [11].

Munteanu et al. proposed electrochemical sensors based on screen-printed electrodes modified with carbonaceous materials (graphene, nanofibers, and carbon nanotubes) and gold nanoparticles. These sensors recorded the cyclic voltammetric profiles of methanol–water (40:10% *v*/*v*) and extracts of extra virgin olive oils, olive pomace oils, and sunflower oils from four different countries, using KCl as an electrolyte. The voltammetric responses, analyzed using PCA and PLS-DA, enabled classification by oil type and geographical origin [56].

Karagozlu et al. utilized the oxidation of alpha-tocopherol on a pencil graphite electrode to differentiate extra virgin olive oil from rapeseed, sunflower, and corn oils, with a detection limit of 10% *w*/*w*. The method involved activating pencil lead using chronoamperometry, immersing the lead in oil for 30 min, drying for another 30 min, and performing differential pulse voltammetric measurements in acetate buffer solution [47]. The lengthy analysis time per sample is a notable limitation of this technique.

## 4. Honey

The potential of electroanalytical techniques for detecting and quantifying honey adulteration lies in the fact that honey contains natural antioxidants, such as flavonoids, phenols, and terpenes, which are electroactive materials. These antioxidants play a natural role in removing oxygen free radicals in the body, a process analogous to the redox reactions on an electrode surface. Consequently, different types of honey, each with unique natural antioxidant profiles, are expected to exhibit distinct electrochemical characteristics [57].

Electroanalytical approaches to addressing honey authenticity primarily focus on detecting adulteration with various syrups and identifying the botanical or floral origin of honey. It is worth noting that certain monofloral honeys are significantly more expensive [58], and that the traditional method for determining botanical origin is melissopalynology, as defined in the EU Council Directive 2001/110.

Honey analysis using voltammetric techniques often requires dissolving the sample in an electrolyte solution (e.g., KCl or phosphate buffer saline solution, PBS) or in specific cases, performing extraction or centrifugation steps [12]. Cai et al. studied the electrochemical behavior of Angelica honey adulterated with rice syrup using cyclic coltammetry on a glassy carbon electrode. They identified twelve variable features (e.g., oxidation and reduction peak potentials, half-peak potentials, and corresponding currents) from the electrochemical signals. These features were analyzed using PCA and LDA to distinguish pure and adulterated samples. Adulteration quantification was achieved using multiple linear regression [57].

Tiwari et al. employed a platinum working electrode to discriminate monofloral honeys based on vοltammetric data analyzed with PCA, LDA, and neural networks to group samples according to floral origin [59]. They also used a carbon paste electrode modified with paraffin oil and ZrO_2_ nanoparticles [60], as well as a carbon paste electrode modified with ZnO nanoparticles [61], for the floral characterization of honey using cyclic voltammetry. Giordano et al. developed a portable device with a gold disk electrode for honey classification based on botanical origin, analyzing voltammetric data with PCA [62]. Guellis et al. combined cyclic voltammetry data obtained on a Cu/CuO electrode with UV-Vis spectrometry for chemometric analysis using PCA and Hierarchical Cluster Analysis (HCA) to differentiate between honey and corn syrup [63]. Wojcik et al. integrated differential pulse cyclic voltammetry performed on a quadruple-disk indium–platinum working electrode with computer vision techniques based on smartphone color recognition to distinguish natural from synthetic honey [64].

Over the past two decades, electronic noses (E-noses) and electronic tongues (E-tongues) have been proposed to classify honey based on botanical or geographical origin, detect adulteration, and identify typical chemical compounds intentionally added to honey [12]. E-tongues are electrochemical analytical devices, comprising single or multi-sensor arrays with cross-sensitivity and low specificity, paired with chemometric tools to establish predictive multivariate statistical models, correlating sensor signals to analytical interpretation. E-tongues can be based on different electrochemical techniques (e.g., potentiometry, voltammetry, and impedance, etc.). Some potentiometric E-tongues may be directly immersed into the honey sample, allowing for direct measurements. In other cases, they may require dissolving a known mass of honey in a specific volume of distilled water [12]. By using potentiometric E-tongue-based devices employing several sensors, the classification of honey according to its floral origin has been achieved [65,66,67,68,69]. Dias et al. introduced a potentiometric E-tongue with 20 cross-selectivity lipid polymeric membranes to detect adulteration with cane sugar, though quantification was not achieved [70].

Voltammetric E-tongues, consisting of multi-working electrodes (e.g., platinum, gold, palladium, copper, glassy carbon, nickel, and silver) paired with a reference and a counter electrode [12], have been employed for botanical origin classification using chemometric techniques [58,66,71] and for detecting adulteration with sugar syrups using PLS regression [13].

Lozano-Torres et al. compared the performance of a voltammetric E-tongue with ^1^H NMR spectroscopy as a reference method for detecting the adulteration of monofloral honey with syrups. Their findings showed comparable results, highlighting the significance of E-tongues as a lower-cost alternative with potential for field measurements [72]. Ciursa et al. developed a cyclic voltammetry-based E-tongue with five working electrodes (gold, silver, copper, platinum, and glass) to detect honey adulteration with different syrups, namely agave, maple, inverted sugar, corn, and rice. Classification was achieved using LDA and SVM, while PLS regression was applied for quantification, though with inferior statistics [73]. Leon-Medina et al. designed an electronic tongue with three working electrodes (carbon, platinum, and gold) to analyze 50 genuine and 50 adulterated honey samples. Following data pre-treatment, classification using K-nearest neighbors (k-NN) achieved 100% accuracy [74].

## 5. Milk and Dairy Products

Electroanalytical techniques have been predominantly used to detect and quantify adulteration in milk and dairy products involving single compounds such as melamine, urea, and hydrogen peroxide. Melamine, in particular, is a commonly used adulterant due to its high nitrogen content (approximately 66% by mass), which can artificially inflate the protein content measured by the conventional Kjeldahl test. However, due to melamine’s low electrochemical activity [75], advanced electrochemical approaches are necessary for its detection.

Rovina et al. demonstrated the detection of melamine in milk products using differential pulse voltammetry (DPV) on a gold electrode modified with chitosan, calcium oxide nanoparticles, and an ionic liquid, achieving an ultralow detection limit [76]. The same group later developed a rapid DPV method for melamine detection in milk powder using a similar electrode modified with chitosan, zinc oxide nanoparticles, and ionic liquids [77]. Heydarian-Dehkordi et al. employed a pencil graphite electrode modified with gold nanoparticles and reduced graphene oxide for melamine detection using cyclic voltammetry [78]. Alternatively, melamine has been voltammetrically detected using an unmodified copper electrode under acidic conditions [79].

Esmaeily et al. introduced a technique involving an electropolymerization-based preconcentration of melamine at a glassy carbon electrode modified with overoxidized poly-(para-aminophenol). During this process, non-electroactive melamine was converted into electroactive poly-(melamine), enabling its quantification through square wave voltammetry (SWV) [80]. El-Shahawi et al. reported an indirect DPV method for melamine detection, utilizing its competition with uric acid as a recognition element on a pre-anodized glassy carbon electrode [81].

Daizy et al. developed a glassy carbon electrode modified with L-arginine and a reduced graphene oxide–copper nanoflower composite to detect melamine via differential pulse voltammetry using ascorbic acid as the active recognition element [82]. Guo proposed an ordered mesoporous carbon/glassy carbon electrode in the presence of copper ions, where melamine was converted to an electroactive Cu-melamine complex, quantified by cyclic voltammetry [83].

Mohebbi et al. fabricated a sensor based on a nanocomposite of poly-orthophenylene diamine (POPD) and graphitic carbon nitride (g-C_3_N_4_), further functionalized with ethylenediaminetetraacetic acid (EDTA) to achieve advanced selectivity for melamine detection via DPV [75]. Feng et al. proposed a nanocomposite of nickel sulfide/nickel oxide and carbon nanotubes for melamine determination in milk using DPV [84]. An et al. employed a screen-printed carbon electrode modified with ferrocenylglutathione (Fc-ECG) for DPV detection, where melamine enhanced the electrochemical signal of Fc-ECG [85].

Molecularly imprinted polymer (MIP) electrochemical sensors, which integrate molecular imprinting techniques with electrochemical detection, have also been developed for melamine detection. Rao et al. modified a glassy carbon electrode with an imprinted membrane and Au/polyaniline composites to enhance selectivity and amplify the electrochemical signal for DPV-based detection [86].

Urea, another nitrogen-rich adulterant used to falsify milk’s protein content, has also been extensively studied. Yu et al. developed an electronic tongue with three working electrodes (Au, Pt, and Pd) combined with multivariate analysis for urea detection in milk [87]. Sha et al. fabricated an amperometric urea sensor by electrodepositing polyaniline on a graphene-modified glassy carbon electrode, achieving a detection limit of 5.88 μM [88]. Ohlsson et al. constructed a sensor using a gold wire working electrode connected via conductive silver epoxy resin to a brass piece, with cyclic voltammetry and Functional Principal Component Analysis (FPCA) demonstrating significant differentiation between adulterated and non-adulterated milk samples (LOD: 85 mg/L) [39].

Shyamala et al. developed a glassy carbon electrode modified with a mixed metal oxide (ZnO/NiO) for the simultaneous detection of melamine and urea in milk using cyclic voltammetry [89].

Hydrogen peroxide, used as an adulterant to extend milk’s shelf life, has also been targeted for detection. Silva et al. utilized batch injection analysis with amperometric detection at a Prussian blue-modified graphite composite electrode for hydrogen peroxide quantification. This approach employed an electronic micropipette to deliver precise sample plugs directly onto the electrode surface, achieving a rapid and proportional electrochemical response [90]. Palsaniya et al. developed reduced graphene oxide (rGO)-MoS₂-modified flexible carbon screen-printed electrodes for real-time amperometric detection of hydrogen peroxide in milk [91]. Shalini Devi et al. reported the co-immobilization of lactoferrin and methylene blue onto a multi-walled carbon nanotube (MWCNT)/nafion-modified glassy carbon electrode for hydrogen peroxide sensing [92].

Detecting the adulteration of expensive milk types, such as goat milk, with less expensive alternatives like cow’s milk, poses unique challenges. Goat milk, prized for its digestibility, nutritional value, and strong antimicrobial properties, is significantly more expensive than cow milk [93]. Demiati et al. employed cyclic voltammetric fingerprinting on a glassy carbon electrode coupled with chemometric data analysis to detect goat milk adulteration with cow milk [94]. Xue et al. used a ten-electrode microsensor array combined with machine learning for voltammetric fingerprinting, achieving a detection limit of 1% for goat milk adulteration in cow milk [95].

The adulteration of fresh milk with reconstituted skim milk powder is less frequently investigated. Nikolaou et al. applied a graphite/SiO₂ hybrid electrode for voltammetric fingerprinting of fresh cow milk, using PCA and class modeling for adulteration identification. Quantification was performed using PLS regression [14].

## 6. Wines

Wine is a complex mixture containing organic species with redox activity. The differentiation of different wines can be achieved via electroanalytical signals attributed to the common phenolic compounds of wines. Carbon-based electrodes, screen-printed graphite/carbon paste electrodes, glassy carbon electrodes, and metallic electrodes are among the most suitable working electrodes for detecting adulteration. Enhancement of sensitivity can be achieved through modification with materials, such as conducting polymers, carbon nanotubes, graphene, and metal complexes [96].

Voltammetric techniques have been employed to study the polyphenolic content (“total polyphenol” or “polyphenol classes”) of wines by measuring the anodic signals (e.g., peak current or area under voltammograms) obtained in characteristic potential intervals [97,98]. Ksenzhek et al. proposed diagrams, called “redox spectra of wines” attributed to the redox behavior of quinones and phenolics, which can be used for the differentiation of different wine samples [98]. Some researchers suggested flow injection analysis (FIA) coupled with amperometric detection at a fixed selective potential for the determination of the polyphenolic content. Thus, electrochemical indexes have been proposed for the evaluation of specific fractions of polyphenols according to specific redox properties. Apart from their use in the assessment of total antioxidant capacity [99] and polyphenol content [100,101], these indexes can be applied for wine authentication. For instance, Sanchez Arribas et al. proposed “electrochemical indexes” corresponding to different polyphenol fractions to group white wines according to their grape varietal [101]. Moreno et al. proposed flow injection analysis coupled with amperometric detection using carbon nanotube/polyvinylpyrrolidone-modified electrodes. Thus, amperometric signals measured at certain potentials were used to differentiate white wine samples according to their grape variety using Principal Component Analysis and Discriminant Analysis [102].

Novakowski et al. coupled cyclic voltammograms recorded on gold and copper working electrodes and treated voltammetric data using Principal Component Analysis to group nine different wine samples (dry red, soft red, dry white, and soft white brands) [4]. Ugliano applied linear sweep voltammetry on disposable screen-printed carbon paste sensors, and using the first order derivative of measured current vs. applied potential, succeeded in the analysis of the main white wine oxidizable compounds and the rapid fingerprinting and classification of white wines from different grape varieties using Principal Component Analysis [40].

Electronic E-tongues have also been proposed for use in the of detection of wine adulteration. Parra et al. [103] successfully developed an electronic tongue based on an array of modified electrodes with different materials to classify red wines and detect adulteration processes. Using a single copper electrode in alkaline medium and taking into account voltammetric measurements at the potential region where copper(II) oxides are generated, Wu et al. [104] were able to differentiate six different brands of Chinese yellow wine, which do not possess constituents that were electroactive at the utilized potential. Schreyer and Mikkelsen [105] were able to differentiate wine samples using a platinum voltammetric electronic tongue, but the proposed sensor was not able to differentiate the origin (Canadian or American wine) or type of wine (white or red wine) in some samples.

Glassy carbon electrodes can also be employed in voltammetric tongues or electrochemical sensors, utilizing a potentiometric panel for the monitoring of wine fermentation and the aging process with the ability to differentiate aged samples stored under different conditions (standard conditions/conditions causing deterioration of the sample quality) [106]. Merkyte et al. proposed an electronic tongue consisting of four GCEs for the amperometric study of six red wines with respect to their antioxidant activity, total phenols content, and bitter taste, and distinguished the considered samples using Principal Component Analysis [107]. Gonzalez-Calabuig et al. developed an electronic tongue consisting of an array of six graphite epoxy-modified composite electrodes to evaluate the metabolites of the Brett defect, 4-ethylphenol, 4-ethylguaiacol, and 4-ethylcatechol using artificial neural networks to build a quantitative prediction model [108]. Voltammetric electronic tongues based on the analysis of several organic acids, such as tartaric, ascorbic, lactic, glutamic, and gallic acid, as well as glucose and tyrosine, have been proposed for wine classification with respect to their origin or aging [109].

Less investigated is the adulteration of white wine with apple juice and apple cider. Liveri et al. used cyclic voltammetry on carbon nanotubes–gold nanoparticle-modified screen-printed electrodes to identify this adulteration, analyzing voltammetric data using class-modeling with a detection threshold of 5% *v*/*v* or lower. The quantification of the adulteration of both apple juice and apple cider was achieved using PLS with acceptable accuracy [7].

## 7. Other Alcoholic Beverages

The adulteration of alcoholic beverages other than wine has been less extensively studied. Novakowski et al. employed cyclic voltammetry on a copper working electrode combined with Principal Component Analysis (PCA) to successfully detect whiskey adulteration with water and glucose and to differentiate between expensive and inexpensive whiskey samples [4]. Similarly, Wojcik et al. utilized differential pulse voltammetry on an iridium quadruple-disk electrode, focusing solely on the faradaic component of the signal. Using the Alternating Trilinear Decomposition (ATLD) algorithm, they were able to distinguish between wines and Scotch whiskies [38].

Zhao et al. demonstrated the differentiation of Chinese liquors based on their aging periods. This was achieved through direct sample analysis using cyclic voltammetry on a gold microelectrode after adding potassium chloride. The voltammetric data were processed with PCA for classification [110].

The authentication of beer has been less extensively studied, as the incentive for adulteration is relatively low due to its modest price. Rosello et al. employed cyclic voltammetry with a carbon screen-printed electrode, using Support Vector Machine Discriminant Analysis (SVMDA) to distinguish between the two primary beer types based on their fermentation processes: ales and lagers. Additionally, Partial Least Squares (PLS) regression and artificial neural networks were used to predict the alcoholic content of the beers [111].

Lvova et al. utilized potentiometric responses from porphyrin-based solvent polymeric membranes to analyze a range of alcoholic beverages, including beer, wine, whiskey, and grappa. The PLS model they constructed successfully predicted the alcoholic content of these beverages [112].

## 8. Fruit Juices

The use of electroanalytical techniques for detecting fruit juice adulteration has been relatively underexplored in the literature [113]. The primary components of fruit juices, aside from water, includes sugars, organic acids, and minerals [19]. Orange and apple juices are the most and the second most produced and consumed fruit juices worldwide, respectively [19,114]. 

Schreyer et al. investigated the dilution of orange juice with phosphate buffer using square wave voltammetry on a platinum working electrode. They analyzed the voltammetric data using PCA and PLS, observing a linear trend [105].

Monago-Marana et al. employed electrochemical fingerprints on disposable carbon screen-printed electrodes to classify different juices, specifically apple, orange, and grape juices, using PLS-DA and PCA-Linear Discriminant Analysis (LDA). The method proved particularly valuable for the verification of the authenticity of orange juice and it was capable of detecting adulteration with grapefruit juice of as low as 1% using PLS-DA [21]. Wojcik et al. proposed the use of differential pulse stripping voltammetry on an iridium quadruple-disk working electrode, aided by deep neural networks, to detect and quantify apple juice adulteration with glucose-fructose syrup [19].

Yu et al. utilized differential pulse voltammetry on a bare glassy carbon electrode to determine the synthetic fluorine dye rhodamine B in fruit juice samples [16].

Salicylic acid is another potential adulterant, often added as a biocide (preservative) to extend the shelf life of juices. Detpisuttitham applied differential pulse voltammetry on a screen-printed electrode to determine salicylic acid in apple juice, as well as in pickled green mustard, pickled bamboo shoots, pickled mango, pickled lime, and pickled ginger [18]. Ahmadi et al. prepared a polymeric nanocomposite (beta-cyclodextrin/arginine) decorated with gold nanoparticle-capped cysteamine. This composite was fabricated on the surface of gold electrodes to create an electrochemical sensor for the determination of azo dyes (carmoisine, sunset yellow, and tartrazine) in orange and pomegranate juices. This method represents a promising platform for the detection and quantification of artificial color adulteration in food products [115].

## 9. Coffee

Coffee is a globally traded commodity with a growing market and increasing interest, particularly in high-quality products from specific origins [38]. The two most popular coffee bean varieties are Arabica (*Coffea arabica*) and Robusta (*Coffea canephora*). Arabica is more expensive, contains less caffeine, and has a more refined taste compared to the sharper, more bitter flavor of Robusta. The highest quality Arabica coffee is classified as premium “specialty” coffee, scoring 80 or above on a 100-point scale, as graded by professional sensory evaluators.

Mutz et al. prepared 10% *w*/*v* coffee extracts in hot water (90 °C) and employed cyclic voltammetry using a 3D-printed graphite–polylactic acid with electrodeposited gold nanoparticles. The voltammetric data were analyzed using SIMCA to detect the adulteration of Arabica coffees from Geographical Indication regions with traditional coffees [38].

Coffee is often adulterated with roasted materials that are difficult to detect visually [22]. De Morais et al. developed a voltammetric electronic tongue consisting of a single carbon paste working electrode to detect the addition of coffee husks and sticks, as well as to assess the shelf life status (i.e., expired or unexpired) of coffee samples. They analyzed 2% *w*/*v* extracts in hot water (90 °C) using square wave voltammetry, with voltammetric data processed via Linear Discriminant Analysis aided by variable selection through a genetic algorithm [23].

Arrieta et al. designed a voltammetric electronic tongue to study the adulteration of Arabica coffee with roasted corn and roasted soybeans. They analyzed 7% *w*/*v* coffee infusions in boiling water using cyclic voltammetry on an array of seven polypyrrole sensors. These sensors were synthesized by the electropolymerization of pyrrole with various doping agents, including sodium sulfate, sodium dodecylbenzenesulfonate (DBS), ammonium persulphate (SF), potassium ferrocyanide (FCN), lithium perchlorate (PC), p-toluenesulfonic acid (TSA), and anthraquinone-2,6-disulfonic acid disodium salt (AQDS), on platinum electrodes. The voltammetric data were analyzed using PCA and PLS [116].

Rodrigues et al. employed an electronic tongue based on a single impedimetric sensor to detect the adulteration of ground roasted coffee (both Arabica and Robusta varieties) with coffee husks and sticks. They analyzed 2% *w*/*v* coffee extracts in hot water (90 °C) using electrochemical impedance spectroscopy on a carbon paste electrode. Chemometric analysis of the impedance spectra data using PLS-DA provided better discrimination than SIMCA [117].

## 10. Future Trends and Perspectives

Electroanalytical techniques, particularly voltammetry, are promising tools for identifying and quantifying food adulteration due to their high sensitivity, rapid analysis, relatively low cost, and potential for field deployment. Key research areas include the analysis of olive oil, honey, meat and seafood, dairy products (e.g., milk, butter, and cheese), spices, alcoholic beverages, fruit juices, coffee, and tea.

Future research in this domain is likely to focus on the development of novel electrodes, such as spark-generated, screen-printed electrodes [118], incorporating materials like metal nanoparticles, graphene, nanotubes, and other nanostructures to enhance sensitivity and selectivity. Biomimetic sensors, created by modifying electrodes with biomimetic compounds to mimic natural recognition elements for specific adulterants, are also expected to play a significant role.

The principles of Green Chemistry are poised to influence this field by promoting the use of eco-friendly and sustainable materials in sensor preparation, developing non-toxic electrolytes and reagents, and minimizing chemical usage in the electroanalytical process to align with environmental considerations. Miniaturization and portability will be key priorities, aiming to develop portable devices [119] and microelectrodes for on-site testing. Wearable voltammetric sensors could further enable real-time analysis of food samples. Ensuring the affordability of such devices is essential to make these technologies accessible, particularly in developing countries.

Machine learning techniques are anticipated to play an increasingly important role in analyzing complex electroanalytical fingerprinting data, including data mining, visualization, discrimination, and class-modeling in order to detect adulteration, while artificial intelligence (AI) could differentiate adulterated samples from authentic ones based on unique electrochemical profiles. Additional contributions of AI filters will include the optimization of working electrode selection, the removal of background noise to improve signal clarity and enhance detection accuracy, and the integration with image recognition to combine voltammetric profiles with visual markers of adulteration, such as unusual color patterns. Another critical challenge is the development of user-friendly interfaces to make these AI-driven tools more accessible to non-experts, including food inspectors and consumers, ensuring widespread adoption and practical application.

For all voltammetric approaches, extensive field studies should be planned and executed using gold standard methods, such as liquid chromatography-mass spectrometry (LC-MS), as reference techniques to validate the accuracy and precision of electroanalytical methods.

## 11. Conclusions

Non-enzymatic electroanalytical techniques provide an effective and innovative solution to the pervasive issue of food adulteration. Their key advantages include high sensitivity, affordability, suitability for field analysis, and potential for real-time detection. The successful application of these techniques across diverse food products has been facilitated by advancements in electrode technology and the integration of chemometric tools, enabling precise adulterant detection. Emerging trends such as machine learning and Green Chemistry principles are poised to further enhance these methods, by providing eco-friendly, scalable solutions that minimize the use of toxic solvents and reduce reagent consumption. However, challenges remain, particularly regarding the complexity of food matrices and the need for extensive validation against gold standard methods. To further advance electroanalytical techniques, efforts should focus on developing comprehensive electrochemical fingerprint libraries for authentic samples (e.g., extra virgin olive oil and monofloral honey varieties), establishing global proficiency testing programs to ensure accuracy and reproducibility, and defining standardized precision requirements, including proposed working electrodes and analytical procedures for different food matrices. Future research should emphasize the development of portable and user-friendly devices, ensuring accessibility for food safety professionals, regulators and, even, consumers. Ultimately, electroanalytical techniques hold immense potential to bolster food authentication, safeguard public health, and promote economic fairness in global markets.

## Data Availability

No new data were created or analyzed in this study.

## Abbreviations

The following abbreviations are used in this manuscript:

AI	Artificial Intelligence
ATLD	Alternating Trilinear Decomposition
DPV	Differential Pulse Voltammetry
EDTA	Ethylenediaminetetraacetic Acid
EIS	Electrochemical Impedance Spectroscopy
EMA	Economically Motivated Adulteration
FPCA	Functional Principal Component Analysis
GCE	Glassy Carbon Electrode
HCA	Hierarchical Cluster Analysis
K-NN	K-nearest Neighbors
LDA	Linear Discriminant Analysis
LOD	Limit of Detection
LOQ	Limit of Quantification
MWCNT	Multi-walled Carbon Nanotube
NMR	Nuclear Magnetic Resonance (NMR) Spectroscopy
PCA	Principal Component Analysis
PLS	Partial Least Squares
PLS-DA	Partial Least Squares-Discriminant Analysis
PLS-LDA	Partial Least Squares-Linear Discriminant Analysis
POPD	Poly-Orthophenylene Diamine
rGO	Reduced Graphene Oxide
RTIL	Room Temperature Ionic Liquid
SIMCA	Soft Independent Modeling of Class Analogy
SVM	Support Vector Machine
SVMDA	Support Vector Machine Discriminant Analysis
SWV	Square Wave Voltammetry
